# Sulfur utilization of corals is enhanced by endosymbiotic algae

**DOI:** 10.1242/bio.020164

**Published:** 2016-08-04

**Authors:** Ikuko Yuyama, Tomihiko Higuchi, Yoshio Takei

**Affiliations:** 1 Center for Information Biology, National Institute of Genetics, 1111 Yata, Mishima, Shizuoka 411-8540, Japan; 2 Graduate School of Science and Technology, Shizuoka University, 836 Ohya, Suruga-ku, Shizuoka 422-8529, Japan; 3 Atmosphere and Ocean Research Institute, The University of Tokyo, 5-1-5 Kashiwanoha, Kashiwa, Chiba 277-8564, Japan

**Keywords:** Endosymbiosis, Skeletogenesis, Coral, *Acropora*, *Symbiodinium*, Sulfate ion

## Abstract

Sulfur-containing compounds are important components of all organisms, but few studies have explored sulfate utilization in corals. Our previous study found that the expression of a sulfur transporter (*SLC26A11*) was upregulated in the presence of *Symbiodinium* cells in juveniles of the reef-building coral *Acropora tenuis*. In this study, we performed autoradiography using ^35^S-labeled sulfate ions (^35^SO_4_ ^2−^) to examine the localization and amount of incorporated radioactive sulfate in the coral tissues and symbiotic algae. Incorporated ^35^SO_4_ ^2−^ was detected in symbiotic algal cells, nematocysts, ectodermal cells and calicoblast cells. The combined results of ^35^S autoradiography and Alcian Blue staining showed that incorporated ^35^S accumulated as sulfated glycosaminoglycans (GAGs) in the ectodermal cell layer. We also compared the relative incorporation of ^35^SO_4_ ^2−^ into coral tissues and endosymbiotic algae, and their chemical fractions in dark versus light (photosynthetic) conditions. The amount of sulfur compounds, such as GAGs and lipids, generated from ^35^SO_4_ ^2−^ was higher under photosynthetic conditions. Together with the upregulation of sulfate transporters by symbiosis, our results suggest that photosynthesis of algal endosymbionts contributes to the synthesis and utilization of sulfur compounds in corals.

## Abstract (precis)

**Summary:** ^35^S-labeled sulfate incorporated into various cells of coral demonstrates that photosynthesis of endosymbiotic algae contributes to the synthesis and utilization of sulfur compounds.

## INTRODUCTION

Reef-building scleractinian corals harbor endosymbiotic dinoflagellate algae of the genus *Symbiodinium* spp. (referred to as zooxanthellae), which provide coral hosts with their photosynthetic products (Muscatine, 1990). Studies using ^14^C-labeled carbon have shown that endosymbiotic algae release photosynthetic products – such as glucose, glycerol and organic acids – that contribute to the coral host carbon requirements (Muscatine and Ceiwichmfu, 1969; Trench, 1971a; Battey and Patton, 1984; Whitehead and Douglas, 2003). Corals also receive various amino acids and fatty acids from symbiotic algae (Trench, 1971b; Wang and Douglas, 1999; Papina et al., 2007). Furthermore, algal photosynthesis results in an increase in the calcification rate in the host skeleton (Al-Horani et al., 2003). Some studies have demonstrated that the incorporation of Ca^2+^ into coral skeletons is accelerated under light conditions and reduced by a photosynthetic inhibitor (Furla et al., 2000; Al-Horani et al., 2003). Thus, endosymbiotic algae are nutrient sources for corals, and their photosynthetic activities may affect coral growth rates.

Few studies have explored sulfate utilization in corals and their symbionts, although sulfur-containing compounds have been detected in coral soft tissues and skeletons. Atomic force microscopic studies have indicated that early mineralization zones, commonly called ‘centers of calcification,’ contain a high concentration of sulfated polysaccharides, and a layered distribution of sulfate has been observed within the crystal-like fibers (Cuif et al., 2003; Cuif and Dauphin, 2005). Sulfated molecules were also detected in the mucus as sulfated sugars, including oligosaccharide side chains of mucus glycoproteins in *Acropora formosa* (Meikle et al., 1987). Another important group of sulfur-containing compounds are the sulfur-containing amino acids (e.g. cysteine and methionine), which play a key role in the synthesis of essential biomolecules, such as antioxidants, vitamins, and co-factors (Saito, 2004). In symbiotic dinoflagellates, the uptake of sulfate and sulfur-containing amino acids has been observed in *Amphidinium carterae*, *A. klebsii*, and *Symbiodinium microadriaticum* (Deane and O'Brien, 1981), suggestive of a sulfate and sulfur-containing amino acid transport system between corals and symbiotic algae. Investigation of the metabolic processing of sulfates in coral will facilitate elucidation of the relationship between corals and algae.

In a previous study, we identified a gene encoding a sulfate transporter as a symbiotically related gene in *Acropora tenuis* (Yuyama et al., 2011). The expression of this gene was consistently upregulated in symbiotic corals compared with aposymbiotic corals (Yuyama and Watanabe, 2008; Yuyama et al., 2011). Immunoreactive sulfate transporters were identified in mucus cells and in the tissue between the coelenteron and skeleton (Yuyama and Watanabe, 2008). These results suggest that sulfate utilization by the coral was enhanced by its symbiotic associations with algae. Thus, exploring which sulfate is utilized by coral is important to understand the endosymbiotic relationship between corals and algae, as well as coral calcification. The purpose of the present study was to determine how corals and endosymbiotic algae utilize environmental sulfate ions. First, autoradiography was performed using ^35^S-labeled sulfate ions (^35^SO_4_ ^2−^) to examine the localization of incorporated sulfate ions. Next, we examined the relative incorporation rate of ^35^SO_4_ ^2−^ into coral and endosymbiotic algae, and their chemical fractions under dark or light (photosynthetic) conditions.

## RESULTS

### Autoradiography

**Fig. 1.**
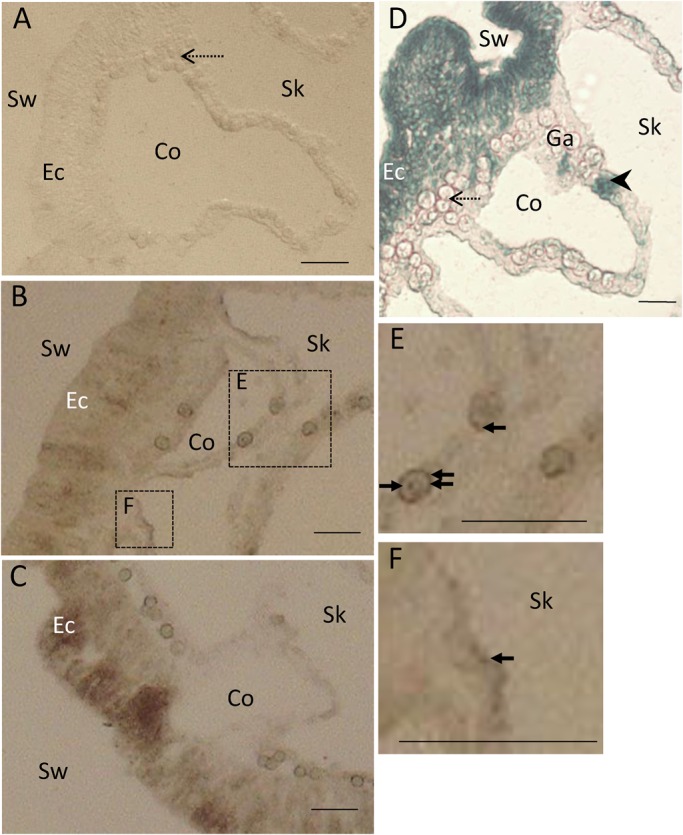
**Light micrographs of decalcified adult coral sections exposed to ^35^S-labeled sulfate ions (^35^SO_4_ ^2−^)**. Coral sections exposed for 0 (A), 6 (B), and 24 h (C), and non-autoradiographic coral sections stained with Alcian Blue (pH 1.0) (D). Areas surrounded by broken lines in B are enlarged in (E,F). Arrows indicate ^35^S grains, arrowheads indicate mucocytes, and the dashed-line arrow indicates an endosymbiotic zooxanthellae. Co, coelenteron; Sk, skeleton; Sw, seawater; Ec, ectoderm; Ga, gastrodermis. Scale bars=20 μm.

After exposure to ^35^SO_4_ ^2−^, incorporated ^35^S was detected as brown dots in the coral sections (Fig. 1). Water-soluble compounds were removed from the coral tissues by dehydration. Therefore, ^35^S-labeled water-insoluble compounds could be detected only in the soft tissues of the coral. Microscopic images of coral sections exposed for 6 and 12 h showed abundant ^35^S grains in symbiotic algal cells, nematocytes and ectodermal cells (Fig. 1B,C,E). In addition, sulfated GAGs were observed in coral tissues by Alcian Blue staining, which was used to detect the presence of mucocytes (Fig. 1D). Ectoderms and mucus cells in the gastrodermis were strongly stained with Alcian Blue, indicating that these cells were mucocytes, and contained sulfated GAGs.

### Measurement of ^35^SO_4_ ^2−^ incorporation into tissue and chemical fractions

**Fig. 2.**
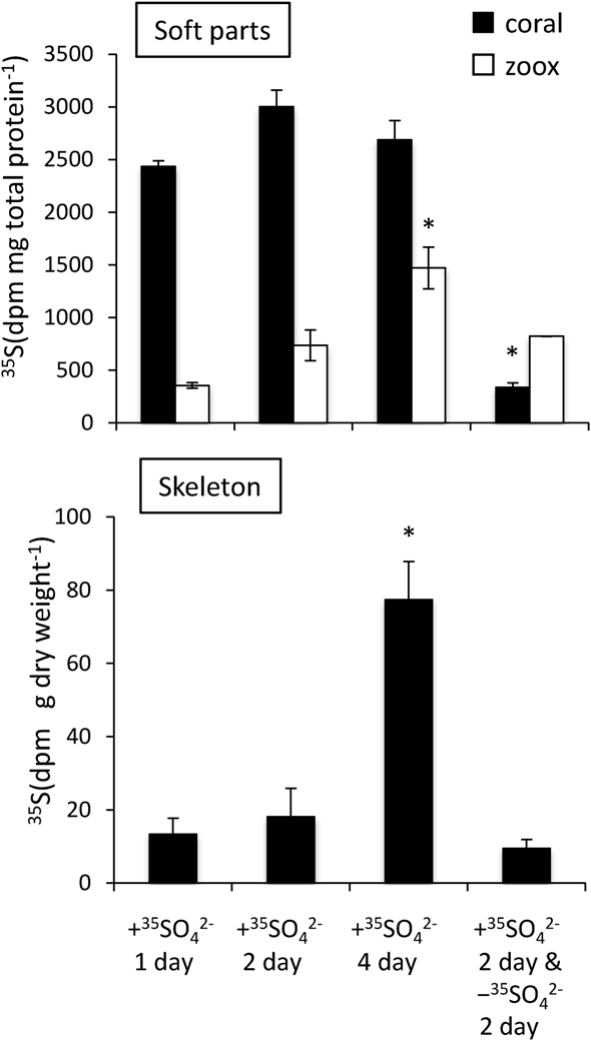
**Incorporation of ^35^SO_4_ ^2−^ into the soft parts of coral and coral skeleton.** Coral tips were incubated for 1, 2, or 4 days in the presence of ^35^SO_4_ ^2**−**^, or in the absence of ^35^SO_4_ ^2**−**^ for 2 days following a 2-day incubation in the presence of ^35^SO_4_ ^2**−**^. Host coral tissues, black bars; zooxanthellae (zoox) cells, white bars. Error bars represent means±standard error (s.e.m.) of biological replicates (*n*=3). *P<0.05 compared with other incubation conditions based on a Tukey–Kramer honestly significant difference (HSD) test.

Incorporation of ^35^S into endosymbiotic algae and coral skeletons tended to increase during the incubation period (*P*<0.05, ANOVA), but the ^35^S quantity in coral soft tissue did not change significantly (Fig. 2). Incorporated ^35^S concentrations in coral soft tissue were generally higher than those in endosymbiotic algae, with the exception of after 2 days of non-exposure (chase). After the 2-day exposure, followed by a 2-day chase period, the ^35^S concentration in endosymbiotic algae did not change significantly with 2-day pulses, whereas that in the coral soft tissue and skeleton decreased by 89% and 48%, respectively, after the 2-day ^35^S exposure (pulse).

**Fig. 3.**
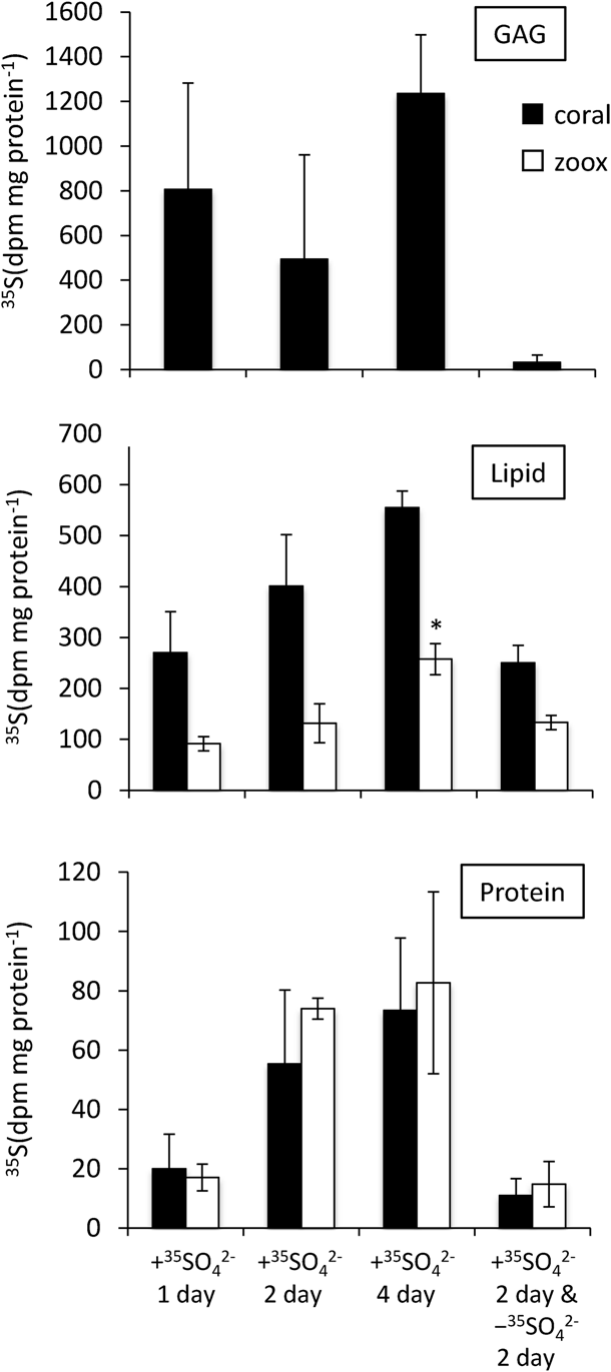
**Incorporation of ^35^SO_4_ ^2−^ into the glycosaminoglycan (GAG), lipid, and protein fractions of coral tissues and zoox cells.** Coral tips were incubated for 1, 2, or 4 days in the presence of ^35^SO_4_ ^2−^, or incubated in the absence of ^35^SO_4_ ^2−^ for 2 days following a 2-day incubation in the presence of ^35^SO_4_ ^2−^. Coral tissues, black bars; zoox cells, white bars. Error bars represent means±s.e.m. of biological replicates (*n*=3). *P<0.05 compared with other incubation conditions based on a Tukey–Kramer HSD test.

Incorporation of radioactivity into the principal chemical fractions of the algal cells and animal tissue is shown in Fig. 3. The distribution of ^35^S radioactivity across the chemical fractions varied significantly between the algae and coral tissue, and showed an increasing trend during incubation experiments. The concentration of ^35^S in GAG fractions was reduced to undetectable levels after the chase. Radioactivity of the lipid fraction after the chase decreased by almost half (62%) that during the pulse period, while the majority remained in algal fractions (101%) after the 2-day pulse. In lipid fractions, the ^35^S radioactivity in coral was 1.8–3.0-fold higher than in the algae lipid fraction, whereas the radioactivity in protein fractions was higher in algae than in corals.

### The effect of light conditions on the incorporation of ^35^SO_4_ ^2−^

**Fig. 4.**
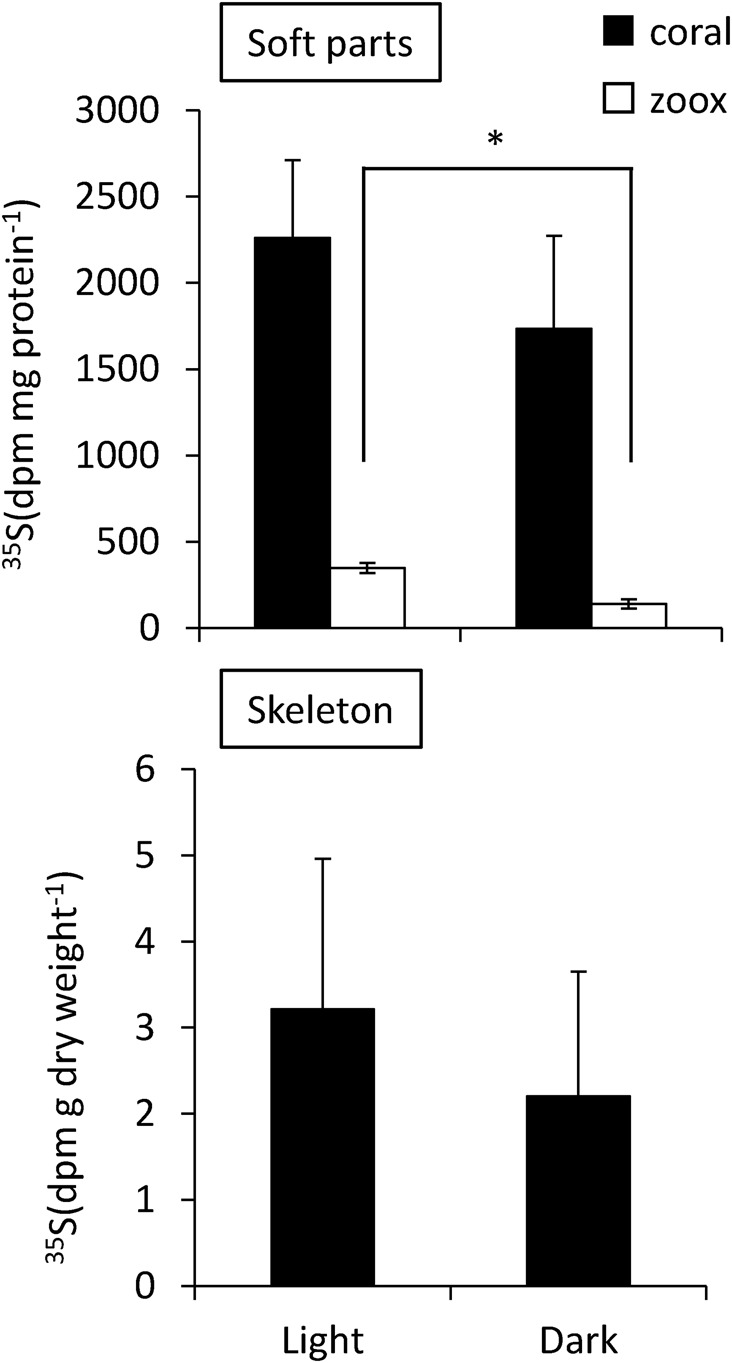
**Effect of light on the incorporation of ^35^SO_4_ ^2−^ into soft parts of coral and coral skeleton.** Coral tips were incubated in the presence of ^35^SO_4_ ^2**−**^ for 12 h under light and dark conditions. Host coral tissues, black bars; zoox cells, white bars. Error bars represent means±s.e.m. of biological replicates (*n*=3). *P<0.05 between light and dark conditions based on a Student's *t*-test.

**Fig. 5.**
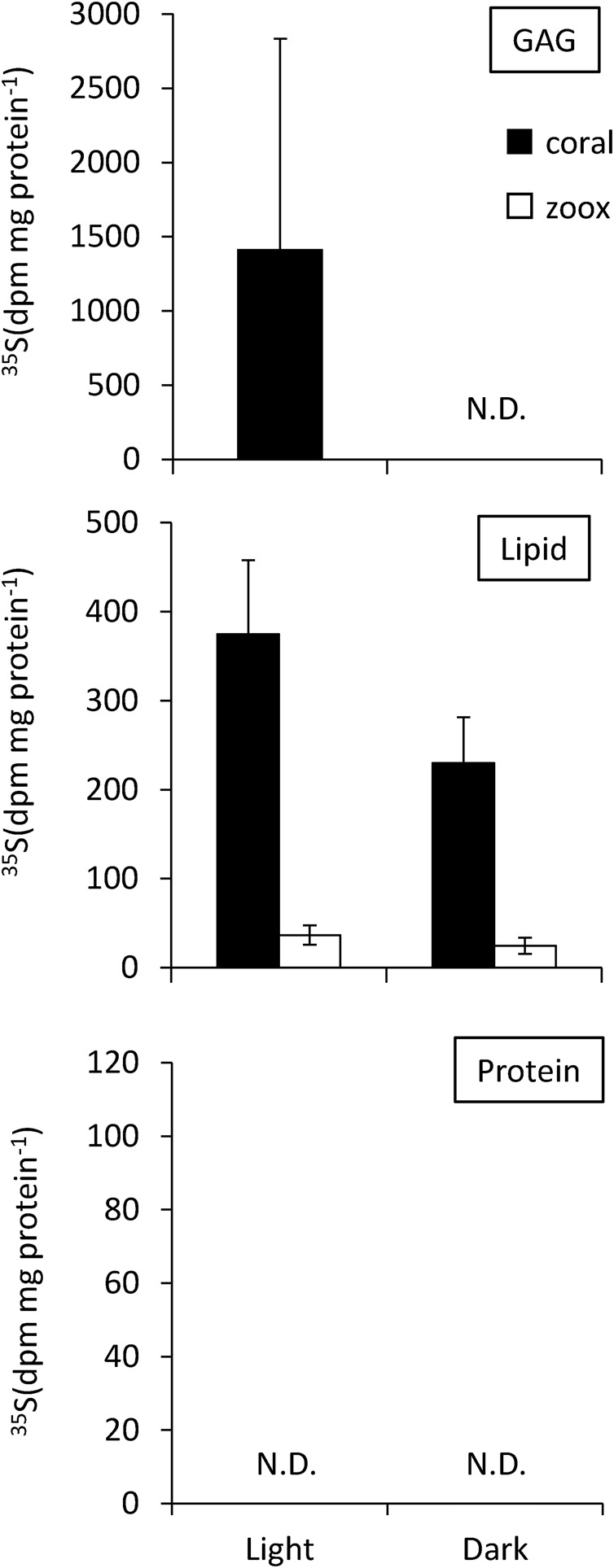
**Effect of light on the incorporation of ^35^SO_4_ ^2−^ into the GAG, lipid, and protein fractions of the coral tissues and zooxanthellae cells.** Coral tips were incubated in the presence of ^35^SO_4_ ^2−^ for 12 h under light and dark conditions. Coral tissues, black bars; zoox cells, white bars. Error bars represent means±s.e.m. of biological replicates (*n*=3).

We also compared the incorporation of ^35^S between light (photosynthesizing) and dark (non-photosynthesizing) conditions (Figs 4, 5). The radioactivity of ^35^S in soft tissue and algae, and their chemical fractions and skeletons, was greater under photosynthesizing conditions than dark conditions. However, significant differences between dark and light were observed only in the algal total fraction, in which the quantity of incorporated ^35^S was significantly higher under light conditions (289, 364, 390 dpm mg protein**^−^**^1^) than dark conditions (96, 131, 188 dpm mg protein**^−^**^1^) (Fig. 4). In the coral GAG fractions, ^35^S radioactivity was detected only in samples incubated under light conditions. The ^35^S concentrations of the protein fractions from 12-h samples could not be estimated since they showed the same level of radioactivity as the reference samples (^35^S unexposed corals) (Fig. 5).

## DISCUSSION

Based on the results of autoradiography using the labeled sulfate ion, we discuss the possibility that the symbiotic relationship with algae affects the synthesis of sulfur compounds in corals.

The incorporated ^35^S content of coral skeleton and endosymbiotic algae increased during the experimental period. In contrast, the amount of ^35^S incorporation into coral soft tissue was not affected by the incubation period. In the light, symbiotic algae incorporated a greater quantity of ^35^SO_4_ ^2**−**^ than in the dark (Figs 1, 4). The incorporated ^35^S was detected around symbiotic algal cells and inside the symbiotic cells (Fig. 1B,E). These results suggest that there is a close relationship between transported sulfate and photosynthesis in symbiotic algae. It was previously hypothesized that endosymbiotic algae promote sulfate utilization of host corals, since higher expression levels of genes coding for a sulfate transporter were found in symbiotic corals compared with aposymbiotic corals (Yuyama and Watanabe, 2008; Yuyama et al., 2011). The results of this study indicate that endosymbiotic algae take up ^35^SO_4_ ^2**−**^ from seawater, and the increased overall incorporation rate of coral tissue under photosynthetic conditions supports this hypothesis. Other elements (C, N and Ca) have been investigated in addition to ^35^S; their rates of incorporation into corals were promoted under light conditions (Muscatine, 1990; Furla et al., 2000; Muscatine and D'Elia, 1978). Thus, endosymbiotic algae provide comprehensive support for the incorporation of various elements by corals.

Autoradiography showed that the transported ^35^S also accumulated in endodermal cells near the skeleton (Fig. 1F). Moreover, the translocation of ^35^S into coral skeletons was confirmed to be influenced by light conditions (Fig. 5). The skeletons of corals are composed, in part, of a polysaccharide, galaxin (cysteine-rich protein), as an organic matrix (Cuif et al., 2003; Cuif and Dauphin, 2005; Tanaka and Ohde, 2007; Fukuda et al., 2003); therefore, sulfate may be incorporated into the polysaccharides that were accumulated in coral skeletons. It is possible that synthesis of these compounds is dependent in part on algal photosynthesis, as described below.

Incorporation of ^35^S sulfate into the GAG fraction increased under light conditions (Fig. 5). A small amount of ^35^S in GAGs were detected after a 2-day non-exposure period, and after 12 h of dark conditions (Figs 3, 5). It is likely that the synthesis of GAGs is affected by algal photosynthesis; furthermore, their metabolic rate is high. Sulfated GAGs were observed in coral tissues by Alcian Blue staining (Fig. 1D). Ectodermal cells, including mucus cells and nematocytes, were strongly stained with Alcian Blue, suggesting that the ^35^S grains in the ectodermal cells were sulfated GAGs from incorporated ^35^SO_4_ ^2**−**^. Considering the synthesis process of sulfated GAGs (Esko et al., 2009), some ^35^SO_4_ ^2**−**^ may have been used for the sulfation of glycoproteins (glycosaminoglycan precursor), and sulfated glycoproteins accumulated in ectoderm cells. The precursor of GAGs is likely derived from algae photosynthesis because the results demonstrated that incorporation of ^35^S into the GAG fractions was strongly affected by light conditions (Fig. 5). However, since the incorporation rate of ^35^S had a large standard error, it is possible that sulfated GAGs are not produced constantly. Based on X-ray analysis, Marshall et al. (2007) showed that sulfur is contained in mucocytes in the coral ectoderm and gastrodermis. Mucus in scleractinian corals is composed of a glycoprotein chain with numerous side chains of sulfated oligosaccharides (Meikle et al., 1987). In addition, sulfated saccharides are present in cnidarian nematocysts (Yamada et al., 2007) and the organic matrix of coral skeletons (Cuif et al., 2003; Cuif and Dauphin, 2005; Tanaka and Ohde, 2007). These oligosaccharides are essential for corals, as a defense against bacteria, and for exchange of materials with the external environment (Brown and Bythell, 2005). Here, the data suggested that some incorporated sulfates are utilized for the synthesis of polysaccharides that contribute to skeletogenesis and mucus production.

^35^S-protein was also detected in both the coral and algal fractions (Fig. 3). These results suggest that sulfate is used to synthesize sulfur-containing amino acids and proteins. A part of the sulfur amino acid synthesis pathway, the sulfur assimilation pathway, is present in plants (Saito, 2004; Leustek and Saito, 1999). The results suggest that the sulfur assimilation pathway is present in endosymbiotic algae, and the resulting sulfur amino acids are translocated into coral tissue. Based on coral genomic information, the cysteine synthesis pathway is absent (Shinzato et al., 2011). To account for this, endosymbiotic algae seem to act as sources of sulfur amino acids for coral. The ^35^S-labeled protein fraction includes products of sulfur amino acids (glutathione and thioredoxin), which play important roles in antioxidant defense (Atmaca, 2004; Battin and Brumaghim, 2009), so that endosymbiotic algae enhance coral defense mechanisms by supplying sulfur amino acids. These results suggest the possibility that coral can utilize components of proteins more efficiently by acquiring endosymbiotic algae.

Sulfolipids are sulfur compounds that accumulate in algae (Garrett et al., 2013), but it remains unclear whether coral can synthesize sulfolipids. We detected ^35^S in the lipid fraction in both coral and algae, and the rate of ^35^S-lipid generation was 1.7-fold higher in corals (143 dpm mg protein**^−^**^1^ day**^−^**^1^) than in endosymbiotic algae (83 dpm mg protein**^−^**^1^ day**^−^**^1^). There was a decrease in the level of ^35^S during the chase period (75 dpm mg protein**^−^**^1^ day**^−^**^1^) in corals, while algal ^35^S lipid levels increased slightly during the chase period (Fig. 3). Thus, ^35^S-labeled lipids in coral tissue were metabolized. In contrast, sulfolipid levels in zooxanthellae were stable during the 2-day period. Although the form in which the compound is accumulated in coral and algae remains unclear, sulfur-containing lipids, such as sulfatide and sulfoquinovosyldiacylglycerol (SQDG), are thought to be included in the ^35^S-labeled lipid fraction. Genes related to the sulfatide biosynthesis pathways have been identified in a coral genome (http://www.kegg.jp/kegg-bin/show_module?T10026_M00067), but not in that of algae (Shoguchi et al., 2013). ^35^S in coral lipid fractions should contain coral-derived lipid sulfatide. Another sulfolipid, SQDG, is a chloroplast membrane lipid (Garrett et al., 2013; Díaz-Almeyda et al., 2011; Awai et al., 2012). Thus, coral and algae contain their own sulfolipids, but whether these sulfolipids are transported between, and utilized by, coral and algae remains unclear. Synthesis of ^35^S lipids in coral and algae increased under light conditions (Fig. 5), suggesting that the ^35^S lipid synthesis pathway is at least in part regulated by algal photosynthesis. Previous studies showed that glucose is one of the lipid precursors transported to the host from algal cells (Whitehead and Douglas, 2003). The photosystem of *Symbiodinium* provides carbon precursors for sulfolipid biosynthesis, which may increase production of ^35^S lipids under light conditions.

Overall, our results showed that (1) coral and their algal symbionts take up sulfate from seawater for the synthesis of organic sulfur compounds, and (2) the incorporation rate of sulfur compounds is higher under photosynthetic conditions, suggesting that the photosynthesis of algal endosymbionts contributes to the synthesis and utilization of sulfur compounds in corals. Endosymbiotic zooxanthellae are required for coral to efficiently utilize sulfur compounds such as sulfur amino acids, sulfolipids and sulfated GAGs. Sulfur metabolism in coral is an intriguing subject, since many sulfur compounds have redox potential (Mitchell, 2003) and are found in coral skeletons (Cuif et al., 2003; Cuif and Dauphin, 2005; Tanaka and Ohde, 2007). In addition, organic sulfur compounds are potential sources of nutrients for coral-associated bacteria (Raina et al., 2013). To further explore the utilization, transport, and metabolism of sulfur in corals, it is important to understand the mechanisms of the coral defense system and coral growth, as well as the coral–algae endosymbiotic relationship.

## MATERIALS AND METHODS

### Biological materials

Adult colonies of *Acropora tenuis* were collected around Sesoko Island in Okinawa, Japan, with permission from the Okinawa prefectural government (No. 24–49). The colonies were transferred to the University of Tokyo or Shizuoka University and maintained in circulated seawater at 22°C under a 12 h light:12 h dark cycle at 100 μmol m^−2^ s^−1^. Coral fragments of about 3 cm in length were prepared by cutting branches from the *A. tenuis* colonies; the top of branches (including some polyps) were maintained for 1 week before the start of the experiment. A total of 30 fragments from four adult colonies were randomly selected for the experiment (*n*=3 per treatment).

### Autoradiography

Branches of adult *A. tenuis* were put into a glass bottle containing 40 ml of filtered natural seawater with (^35^S)-sodium sulfate. The (^35^S)-sulfate (370 MBq ml^−1^; GE Healthcare, Little Chalfont, UK) in a total volume of 6 μl was added to filtered natural seawater. The water temperature was set at 22°C; the incubation times were 6 and 24 h under light conditions (100 μmol m^−2^ s^−1^). After incubation, the coral tip was excised from the plates and washed three times with sterilized seawater. Unexposed coral branches (exposure time to ^35^S-labeled sulfate, 0 h) were prepared as the experimental control. Branches were transferred to glass bottles containing 40 ml of filtered natural seawater (without addition of ^35^S) as the experimental treatment. The coral samples were fixed, decalcified and embedded in Paraplast^®^ as described by Yuyama and Watanabe. (2008). De-paraffinized coral tissue sections were then dehydrated through a graded ethanol series (70, 90, and 100%) containing 0.3 M ammonium acetate and air-dried. Thereafter, the sections were dipped in Hypercoat Emulsions (GE Healthcare) and exposed for 3 days in darkness. After development with Kodak D-19 developer (Rochester, NY, USA) and fixation, they were dehydrated. Microscopic observations of the coral sections were performed using a stereomicroscope (SZX-ILLK 100; Olympus, Tokyo, Japan).

### Alcian Blue staining

Coral sections not exposed to ^35^SO_4_ ^2−^ were stained with Alcian Blue to detect sulfated glycosaminoglycans (GAGs). Fixation, decalcification, and embedding were performed as described above. After de-paraffinization, the sections were placed in 0.1 N HCl for 5 min and then stained with 1% Alcian Blue (pH 1.0) for 30 min (Jones and Reid, 1973). Microscopic observations of these sections were made using a stereomicroscope (SZX-ILLK 100; Olympus).

### Quantification of incorporation of ^35^S-labeled sulfate

Branches of adult *A. tenuis* were placed in a glass bottle containing 20 ml of filtered natural seawater with (^35^S)-sodium sulfate. The (^35^S)-sulfate (370 MBq ml^−1^; American Radiolabeled Chemicals Inc., St. Louis, MO, USA) in a total volume of 3 µl was added to filtered natural seawater. The water temperature was set at 22°C under a 12 h light:12 h dark cycle at 100 µmol m^−2^ s^−1^. In the first experiment, branches were incubated for 1, 2 or 4 days. In the second experiment, branches were incubated in filtered natural seawater for 2 days after a 2-day incubation with (^35^S)-sodium sulfate. In the third experiment, branches were incubated for 12 h under light (100 μmol m^−2^ s^−1^) and dark conditions. Replicate groups of three branches were used for each treatment. After incubation, the coral tip was washed three times with fresh seawater. An unexposed coral branch (exposure time to ^35^S-labeled sulfate, 0 h) was prepared as the experimental control. The counts in the samples incubated without added radioactivity were subtracted from the values of the experimental samples.

### Measurement of ^35^S in endosymbiotic zooxanthellae, coral soft tissue, and skeleton

Soft tissue was removed by using the water-pick method, which stripped the tissue from the coral skeleton into approximately 30 ml of 100 mM phosphate buffer with 10 g liter^−1^ NaCl (pH=7.0). Removed tissues were homogenized and centrifuged (twice at 600×***g*** for 10 min) to separate the supernatant (coral tissue) and pellets (zooxanthellae). The animal tissue fraction was adjusted to a known volume and the algal pellet was resuspended in 2 ml of 100 mM phosphate buffer with 10 g liter^−1^ NaCl. The protein content of each homogenate was quantified by Bradford assay (Bradford, 1976). Portions of the algae and coral fractions were re-suspended in hot 80% alcohol and adjusted to a known volume, after which radioactivity was evaluated using a liquid scintillation counter (LSC-5100; Aloka, Tokyo, Japan). The counting efficiency (cpm/dpm ratios) for ^35^S was calculated to be 0.7. ^35^S content in seawater was adjusted to 1×10^4^ dpm ml^−1^ for all experiments. Skeletons were boiled in 6 N NH_4_OH to dissolve and remove residual animal tissue and algae. The tissue-free skeleton was washed in distilled water and the weight of the dried skeleton was measured using an electronic balance. No detection of ^35^S was confirmed in distilled water for final washing. The clean skeleton was dissolved in cold 6 N HCl, and the HCl fraction containing the ^35^S was counted after filtration by 0.45 µm PTFE filter.

### Fractionation of ^35^S labeled components of corals and zooxanthellae

Coral and algae fractions were divided for preparation and enumeration into the trichloroacetic acid (TCA) insoluble (protein) fraction (Whitehead and Douglas, 2003), lipid fraction (Bligh and Dyer, 1959), and GAG fraction (Zhang et al., 2001). Samples of the algal and host coral fractions were fixed in 10% TCA for 15 min and then centrifuged at 10,000×***g*** for 15 min. The supernatant was washed with 100% ethanol and then resuspended in 500 μl of 8 M guanidine HCl to detect ^35^S using a liquid scintillation counter. The lipid fraction was extracted from the coral and algae tissue fractions with 1 ml methanol: chloroform (2:1) for 15 min at room temperature, followed by centrifugation at 1500×***g*** for 15 min. The chloroform layer containing the lipids was separated from the methanol layer by the addition of 0.5 ml of 0.1 M KCl. The chloroform layer was used for counting ^35^S. To prepare the GAG fraction, an Alcian Blue assay was performed as described by Frazier et al. (2008). The resulting pellet was dissolved in 500 μl of 8 M guanidine HCl and assessed using a liquid scintillation counter. The experiments were performed in triplicate. The GAG fraction was not prepared from the algae fraction because endosymbiotic *Symbiodinium* in coral tissue sections were not stained by Alcian Blue. Counts in the fractions incubated without added radioactivity were subtracted from the values of the experimental samples.

### Statistical analysis

All data were normalized to protein content or skeleton dry weight. Statistical analyses were performed using JMP software (v. 8.0; SAS Institute, Cary, NC, USA); the normalized values were compared with an analysis of variance (ANOVA). *Post hoc* differences were evaluated using the Tukey–Kramer honestly significant difference (HSD) test.
